# An assessment of soybeans and other vegetable proteins as source of salmonella contamination in pig production

**DOI:** 10.1186/1751-0147-52-15

**Published:** 2010-02-17

**Authors:** Martin Wierup, Per Häggblom

**Affiliations:** 1 Department of Biomedical Sciences and Veterinary Public Health, Box 7009, Swedish University of Agricultural Sciences, 75007 Uppsala, Sweden; 2 Department of Chemistry, Animal feed and Environment, National Veterinary Institute, 75189, Uppsala Sweden

## Abstract

**Background:**

The impact of salmonella contaminated feed ingredients on the risk for spreading salmonella to pigs was assessed in response to two incidences when salmonella was spread by feed from two feed mills to 78 swine producing herds.

**Methods:**

The assessment was based on results from the salmonella surveillance of feed ingredients before introduction to feed mills and from HACCP - based surveillance of the feed mills. Results from the mills of the Company (A) that produced the salmonella contaminated feed, were by the Chi. Square test compared to the results from all the other (B - E) feed producers registered in Sweden. Isolated serovars were compared to serovars from human cases of salmonellosis.

**Results:**

Salmonella (28 serovars) was frequently isolated from imported consignments of soybean meal (14.6%) and rape seed meal (10.0%). Company A largely imported soybean meal from crushing plants with a history of unknown or frequent salmonella contamination. The risk for consignments of vegetable proteins to be salmonella contaminated was 2.4 times (P < 0.0006) larger for A when compared to the mills of the other companies which largely were supplied by soybean meal from a crushing plant with a low risk for salmonella contamination. Also the level of feed mill contamination of salmonella was higher for feed mills belonging to Company A in comparison to the other companies before and also after heat treatment. Four (10.5%) of the 38 serovars isolated from feed ingredients (28) and feed mills (10) were on the EU 2007 top ten list of human cases of salmonellosis and all but eight (78.9%) on a 12 year list (1997-2008) of cases of human salmonellosis in Sweden.

**Conclusions:**

Salmonella contaminated feed ingredients are an important source for introducing salmonella into the feed and food chain. Effective HACCP-based control and associated corrective actions are required to prevent salmonella contamination of feed. Efforts should be taken to prevent salmonella contamination already at the crushing plants. This is challenge for the EU - feed industry due to the fact that 98% of the use of soybean/meal, an essential feed ingredient, is imported from crushing plants of third countries usually with an unknown salmonella status.

## Background

In the EU, salmonellosis and campylobacteriosis are the most frequently occurring zoonotic infection in humans. Up to approximately 200 000 human cases are annually reported for each of these infections in the EFSA zoonoses reports since 2004 [1]. Other remaining zoonoses are reported to occur in much lower numbers, approximately: Yersiniosis (9000 cases), VTEC (2900), Listeriosis (1500), Echinococcosis (800), Tricinillosis (800), Brucellosis (500), Tuberculosis caused by M. bovis (120) and rabies (<5). The majority of the food borne outbreaks reported in EU are also caused by salmonella and e.g. of the food borne outbreaks reported during 2005 (5311 outbreaks involving approx. 47251 human cases out of which 5330 hospitalised and 24 deaths) salmonella was the most important causative agent (64%), followed by Campylobacter (9%) and viruses (6%) [2].

In order to decrease the burden of salmonella infections focus have been given to the prevention of the introduction of salmonella into the food chain through the food animal production. In the EU, actions were primarily directed to poultry producing eggs and meat. Currently actions are planned to bring down the prevalence of salmonella contamination in the swine production in accordance with Regulation (EC No 2160/2003). So called baseline studies have been performed in all Member States to obtain a more comparable estimate of the prevalence of contamination in the Member States [1]. In addition, EFSA has conducted different risk assessments on how to reduce the prevalence of salmonella in the swine production [3] as a base for those target levels for salmonella contamination that the EU Commission will set according to the new food law ((EC) No 178/2002) that is in power since 2002. In this respect attention has also been given to the importance of salmonella contamination of animal feed as being the first link of the animal derived food chain [4,5].

A striking example of the potential of animal feed as a source of salmonella infections in humans occurred when *S*. Agona emerged as a public health problem in several countries due to the spread of contaminated imported fish meal used in animal feed. In the United States a rapid increase of human infections with *S*. Agona occurred from 1968 to 1972 [6] and a similar increase of human infections with *S*. Agona occurred simultaneously in European countries. Since then, S. Agona is among the most prevalent serotypes in humans e.g. in the USA, and it is estimated that the serotype has caused more than one million human illnesses in the USA alone since it was introduced into the food chain [7].

It is difficult to evaluate the importance of feed as a source of salmonella infection in animals and its subsequent spread to humans, when several serovars of salmonella simultaneously occur in different parts of the food chain, which currently is the case in many Member States of the EU [1]. In contrast it is easier to perform such an assessment in a country with a low prevalence of contamination in the animal food chain.

Against this background we present an assessment of the possible impact of salmonella contaminated vegetable protein as feed ingredients on the risk for spreading salmonella in animal feed production in Sweden, a country which has demonstrated a very low prevalence of salmonella in the animal food l production [1] and where data since many years are available from the control of the feed production [4]. The study was done during a two year period when salmonella was spread by feed from two feed mills to 49 and 29 swine producing herds respectively [8,9]. Both feed mills belonged to the same company (A).

The salmonella contamination of high risk feed materials, when tested before introduction to the feed mills, is presented as well as the result of HACCP-based control of salmonella contamination at different sampling points in the production environment within the feed mills. The results from Company (A) were compared with results from all other commercial feed mills in Sweden during the same period. In addition, the results from of the HACCP control of feed mills from 2000 to 2005 as well as from two randomly selected earlier years (1991 and 1997) is presented. The study is based on an inquiry on behalf of Swedish Board of Agriculture [20].

## Methods

### 1. Feed mills

The Swedish feed industry has undergone significant changes to meet the rationalization in the farm sector characterized by a dramatic decrease in number of food animal producing enterprises and to an overall decrease of the feed volume produced by ca 15% during the last 10 years. The largest company (A) was operating 9 feed mills, while Company B which was running two factories and was joined to A by a business agreement. Company C was a group running 4 feed mills. Company D owned one feed mill and those under E comprised several smaller enterprises. The relative volume of feed produced by each group is indicated by their estimated market share as presented in Table 1. During the period of the study the mean size of Swedish feed mills (volume produced per feed mill) was the second largest of the EU member states according to European Feed Manufacturers Federation [10]. Pig feed is in most cases produced in the same feed mill as poultry feed, however, pig feed may also be produced in feed mills where cattle feed is produced. The number of feed mills producing pig feed was for Company A 5, B 1 and C 2. The number of production lines varies between the feed mills.

**Table 1:** Number of salmonella positive samples from the weekly environmental surveillance of the production line of feed mills in Sweden - before heat treatment; and distribution of positive samples and estimated share of national feed production between 1995 and 2005

Year/Feed producing company	No. of salmonella positive samples before heat treatment (% distribution of positive samples between companies for each year)									Estimated share of national production
		
	1995	1997	2000	2001	2002	2003	2004	2005	Total	
**A +B**	36 (90%)	22 (82%)	45 (94%)	20 (95%)	17 (94%)	21 (66%)	7 (88%)	19 (90%)	187 (87%)	75%

**C**	4 (10%)	5 (18%)	2 (4%)	1 (5%)	1 (6%)	7 (22%)	0 (0%)	2 (10%)	22 (10%)	15%

**D+E**^1)^	-	0 (0%)	1 (2%)	0 (0%)	0 (0%)	4 (12%)	1 (12%)	0 (0%)	6 (3%)	10%

**Total**	40	27	48	21	18	32	8	21	215	100%

1) Includes also technically less advanced smaller factories

### 2. Control of high risk feed material

According to national legislation (SVS 2006:81) feed materials are categorized according to risk for salmonella contamination, and those feed ingredients found to have a high risk have to be tested negative for salmonella contamination before being used for feed production. In practice they are not allowed to enter the feed mill before a negative test result are at hand. Consignments found to be salmonella contaminated are subjected to a decontamination procedure by using organic acids followed by re-testing with negative result. During the period studied, the high risk feed materials used for feed production intended to swine, involved soybean meal, rape seed meal, palm kernel meal and maize/corn. The products were imported to Sweden by the feed companies. Each consignment was usually a shipload of 1200 - 2500 tonnes or trucks loading 30 tonnes.

### 3. HACCP control in feed mills

In line with the EU legislation, (EC) No 2160/2003 on control of salmonella and other zoonotic agents, a national control programme for feed was established in Sweden 1993 (although not yet a harmonized demand in the EU). In this legislation (SJVFS 2006:81) the following five critical control points in the processing line were identified in feed mills manufacturing compound feed for food producing animals:

1. Top of bin for final feed (compound feed).

2. Room for pellet coolers.

3. Top of pellet cooler.

4. Dust from the aspiration system (filter).

5. Intake pit/bottom part of elevator for feed materials.

At these critical control points dust samples or sweepings are collected.

When poultry feed is produced, a minimum of one environmental sample has to be taken at each of the above five critical control points on a weekly basis and checked for the absence of salmonella. When only non-poultry feed is produced the corresponding requirement is limited to control points 1 and 5. In addition to surveillance of the processing line, heat treatment of poultry feed is a requirement in the legislation. These samples are taken by the operator and all samples have to be sent to the National Veterinary Institute (SVA) for analysis and control that the number of samples is in accordance with the legislation. However, most operators normally take additional environmental samples on a voluntary basis. When salmonella is detected further serotyping is carried out.

The national legislation also prescribes the actions to be taken when salmonella is found in feed mills. According to the legislation the competent authority has to be notified when salmonella is isolated after heat treatment, and depending on location in the feed mill and feed type the actions varies from further sampling to assess the problem to immediate stop of production.

Cleaning and disinfection as well as follow up procedures are always carried out according to a plant specific cleaning programme that has to be in place. When justified the competent authority (Swedish Board of Agriculture) can modify these programmes.

### 4. Sampling and bacteriological methods

The surveillance of feed ingredients is based on a sampling procedure which takes into consideration an uneven distribution of salmonella contamination and is designed to detect a contamination in 5% of the batch with 95% probability [11]. The size of the analytical sample is 25 gram and usually 8 samples are analyzed; each consisting of 10 pooled subsamples of 2.5 gram.

The detection of salmonella from feed ingredients is based on culture methods according to the NMKL-71 method [12]. The same method is also applied for the analyses of samples in the HACCP control. Samples taken when positive samples are identified can also be analyzed at other approved laboratories. Environmental samples taken in addition to the legislation are usually analyzed by industry laboratories.

In order to obtain information whether feed associated serovars were identical to serovars in human cases of salmonellosis a comparison was done with EFSA ten most prevalent serovars from human cases of salmonelosis in the EU (EFSA 2009). The data were also compared with corresponding data from Sweden between 1997-2008 covering all serovars of salmonella reported and subtyped by The Swedish Institute for Infectious Disease Control (Ivarsson 2009; personal communication).

### 5. Statistical analyses

Statistical analyses were carried out by the Chi. Square test [13].

## Results

### Imported high risk feed material

**Figure 1:**
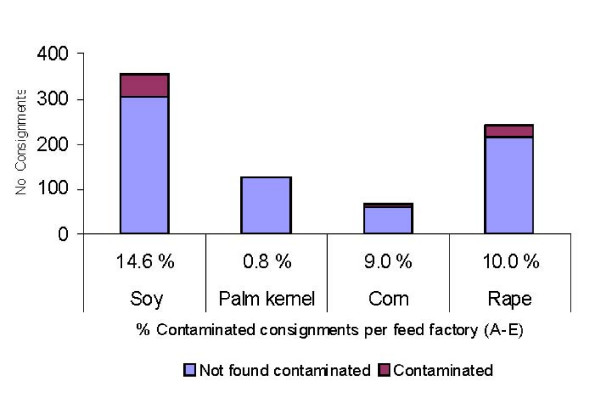
**Salmonella contamination of feed proteins imported to Sweden by feed factories 2004-05**. Number of consignments of vegetable feed ingredients imported to Sweden from non Scandinavian sources during 2004-2005 and percent of these found contaminated by salmonella in control before introduction in feed mills.

**Figure 2:**
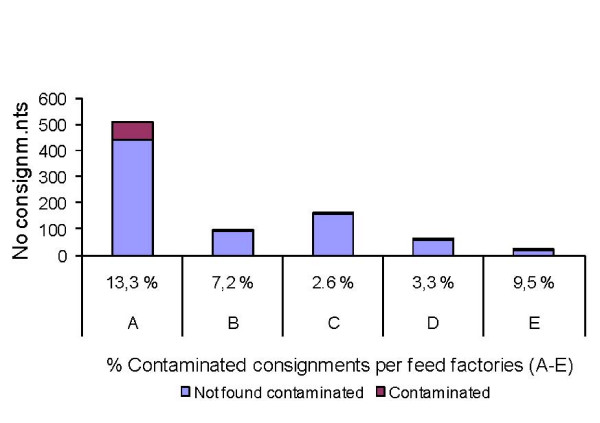
**Salmonella contamination of feed proteins imported to Sweden by feed factories 2004-05**. Total number of consignments of vegetable feed ingredients imported to Sweden (including from Scandinavian sources) by different feed producing companies during 2004-2005 and the proportion found contaminated by salmonella in control before introduction in feed mills.

In 2004 and 2005 a total of 795 (year/no consignments: 2004/398 and 2005/397) consignments of vegetable proteins, mostly soybean and rapeseed meal, were imported to Sweden. A total 5250 pooled samples were investigated for salmonella contamination and 131 (2.5%) of the samples and 83 (10.4%) of the consignments were contaminated. When result was split into different products 14.6% and 10.0% of imported consignments of soybean meal and rapeseed meal, respectively, were found to be contaminated (Figure 1). It should be noted that Figure 1 in contrast to Figure 2, does not include soybean meal which was tested negative for salmonella before export from a Scandinavian source, at a volume corresponding to the mean size of approximately 46 shiploads.

The soybean products were imported either from a Scandinavian crushing plant with a long term documented quality assurance for freedom of salmonella contamination or from different producers in South America, usually from Brazil The latter soybean meal was imported by two feed producing companies (A and B) and the salmonella contaminated consignments shown in Figure 2 were also concentrated to these two companies. The difference between A and B is likely to reflect that they used soy or rape seed meal from different producers. In 2004 Company A imported the majority of the soybean meal from South America and 29 out of 144 imported consignments were contaminated by salmonella (20.1%) based on the sampling methods used.

In 2004 54% of the investigated consignments were imported by Company A and contained 90% (p < 0.0001) of the salmonella contaminated samples taken from the vegetable proteins imported, and 71% of the serovars (p < 0.01). In 2004 16.0% and in 2005 10.7% of the consignments imported by A were found to be salmonella contaminated. The risk for imported consignments to be salmonella contaminated in both 2004 and 2005 was 2.4 times (P < 0.0006) larger for consignments imported by A than by the other importing feed mills.

### 2. HACCP control within feed mills

The result of the mandatory environmental samples taken at different control points before and after heat treatment is presented in Table 1 and 2. In addition to 2004-2005, Table 1 and Table 2 also include results from a ten year period to get a wider perspective of the contamination rate. In these tables all feed mills belonging to Company A also includes Company B because of the business agreement that started 2000) and because data were initially presented as a total. However, Company B operated its HACCP control as before that agreement which is highlighted below. The proportions of salmonella contaminated samples from feed mills from A and B before and after heat treatment (87%; Table 1 and 86%; Table 2 respectively), were larger than expected by their market share (75%), during all the ten years studied. In contrast, the opposite situation was found for the feed mills belonging to C-E. The results from the samples taken after heat treatment were of particular interest. None of those samples from the factories belong to the C group were found to be contaminated. The salmonella contamination after heat treatment in group D included non traditional small feed mills some of which closed down as a result of sanitation procedures following the contamination.

**Table 2:** Number of salmonella positive samples from the weekly environmental surveillance of the production line of feed mills in Sweden - after heat treatment; and distribution of positive samples and estimated share of national feed production between 1995 and 2005

Year/feed producing company	No. of salmonella positive samples after heat treatment									Estimated share of national production
		
	1995	1997	2000	2001	2002	2003	2004	2005	Total **(%)**^2)^	
**A + B**	7	7	7	1	2	7	0	1	32 (86%)	75%

**C**	0	0	0	0	0	0	0	0	0 (0%)	15%

**D+E**^1)^	1	0	0	1	2	1	0	0	5 (14%)	10%

**Total**	8	7	7	2	4	8	0	1	37 (100%)	100%

1) Include also non - traditional" smaller factories

2)% distribution of positive samples between companies for each year

As a further elucidation of the efficiency of the HACCP control, data from A and B feed mills were separated (Tables 3 and 4) and studied 2000 - 2005. It can be seen that salmonella contamination before heat treatment occurred in factories of both companies (Table 3). Feed mill of B periodically faced severe problems (years 2000, 2003 and 2005) with an in house contamination of unknown origin, when up to 14 different serovars of salmonella where detected per period. At Company B, in contrast to A this contamination was not detected after heat treatment (Table 4).

**Table 3:** Number of salmonella positive samples from environmental weekly surveillance of the production line - before heat treatment of feed mills of A and B using largely the same feed ingredients

Year/feed producing company	Number of salmonella positive samples collected before heat treatment						Total
		
	2000	2001	2002	2003	2004	2005	
**A**	32	17	14	10	7	3	83

**B**	13^1)^	3	3	11^2)^	0	16^3)^	46

1. Nine different serovars of salmonella isolated.

2. Nine different serovars of salmonella isolated.

3. 14 different serovars of salmonella isolated.

**Table 4:** Number of salmonella positive samples from environmental weekly surveillance of the production line - after heat treatment of feed mills of A and B using largely the same feed ingredients

Year/feed producing company	Number of salmonella positive samples collected after heat treatment						Total
		
	2000	2001	2002	2003	2004	2005	
**A**	7	1	2	7	0	1	18

**B**	0	0	0	0	0	0	0

**Figure 3:**
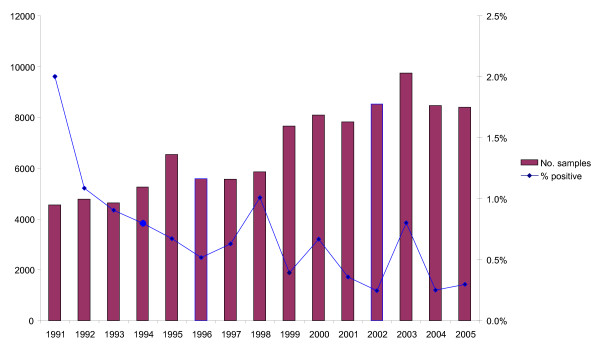
**Number of environmental samples of the production line (HACCP - control) taken voluntarily (1991-1992) and according to legal requirements (1993-2005) for salmonella surveillanceof registered/approved feed mills in Sweden**.

In Figure 3 is the summary result of environmental surveillance for salmonella contamination (HACCP) taken in Swedish feed mills from 1991 to 2005. The figure also includes samples taken as a follow up procedure when salmonella contamination was identified. In spite that the commercial feed production roughly has decreased by approximately 10% during the period the number of samples has increased from 4000 to 8000 samples per year. During the same period the proportion of salmonella positive samples had decreased from 2% to < 0.5%.

### Salmonella serovars

A total of 28 different serovars of salmonella, including one non typeable strain, were isolated from vegetable proteins imported to Sweden during 2004-2005. These serovars were in alphabetic order: *S*. Adelaide,*S*. Agona,*S*. Anatum,*S*. Bere, *S*. Cerro, *S*. Cubana, *S*. Gaminara, *S*. Glowcester, *S*. Havana, *S*. Infantis, *S*. Kentucky *S*. Lexington, *S*. Livingstone,*S*. Llandoff, *S*. Mbandaka, *S *Morehead, *S*. Muenster, *S*. Obogu, *S*. Ohio, *S*. Panama, *S*. Reading,*S*. Rissen, *S*. Senftenberg, *S*. Tennessee, *S*. Tabligo, *S*. Typhimurium (not phage typed) and *S*. Yoruba. When adding those serovars isolated in 2000, 2003 and 2005 in the HACCP control in the feed mill of Company B (Table 3, data from the remaining factories and years not present) an additional 10 new serovars were isolated. These were: *S*. Bredeney, *S*. Corvallis, *S*. Glostrup, *S*. Kingston, *S*. Schwarzengrund,*S*. Typhimurium phage type 41, *S*. Typhimurium phage type 193, *S*. Oranienburg, *S*. Umbilio and *S. Ouakam*.

Four (10.5%) of the serovars isolated (*S*. Agona,*S*. Infantis, *S*. Kentucky and *S*. Typhimurium-included at least two different strains) were identical to the 10 most common isolates of human cases of salmonellosis in the EU [1] and out of the 38 feed associated serovars identified 78.9% or all but 8 (S. Bere, S. Glowcester, S. Llandoff, S. Morehead S. Obogu, S. Ouakam,, S. Tabligo and S. Yoruba) had been isolated from human cases of salmonellosis diagnosed in Sweden 1997-2008.

## Discussion

Salmonella was frequently isolated from consignments of vegetable proteins used as feed ingredients, in particular from soybean meal (14.6%) and rape seed meal (10.0%) (Figure 2). When the majority of the imported soy was from South America 20.1% of the consignments were contaminated by salmonella (data not shown). Even higher levels, up to 30%, have regularly been found in the Sweden when high risk ingredients from South America are tested before introduction to the feed mills [14]. The frequent isolation of salmonella from vegetable proteins is in agreement with several observations from different countries. In a Dutch report 3.2% and 6.7% of Brazilian extracted soybeans were found positive for salmonella during 2002 and 2003 respectively [15]. In a recent comprehensive study based on an annual examination of up to 80,000 lots of feed, Kwiatek et.al [16] report that in Poland up to 15.0% and 15.4% of imported lots of soy and rape seed were respectively salmonella contaminated in 2005-2007. Corresponding data for products produced in Poland were 6.3% and 7.7%. In summary, it can be concluded that the oil seed feed ingredients are often contaminated by salmonella although it is difficult to compare the level of contamination between different studies because the results depend on the sampling and culture techniques applied.

The study also demonstrates that the vegetable proteins used by Company A significantly more often were salmonella contaminated, and also harboured a significantly larger number of different serovars, than feed ingredients used by the remaining companies. This reflects that Company A largely had replaced a supplier of soy products with a high level of quality control for freedom of salmonella with imports from South America. This was the case particularly in2004 when 29 out of 144 imported consignments were contaminated by salmonella (20.1%, data not shown). As a comparison the mills of Company C, which bought all soy products from a supplier with a high salmonella quality assurance, none (0%) of the imported 46 consignments were salmonella contaminated during that year (data not shown).

The higher exposure to salmonella by feed ingredients to feed mills of Company A, in comparison to the mills of Company C - E, was reflected in an increased in house contamination of salmonella of the mills of Company A, also when including Company B. The contamination was detected before the heat treatment (Table 1) but more severe also after that step (the clean area). No salmonella contamination was detected after heat treatment Company C feed mills in spite the fact that salmonella contamination occurred before that step.

The results from the feed mills of Company B are of special interest. In spite of periods with heavy salmonella contamination during three years (2000, 2003 and 2005) before the heat treatment process, the HACCP surveillance could never detect any salmonella contamination after that step in contrast to in the feed mills of Company A (Table 3 and 4). This demonstrates that management procedures, supported by HACCP-control and heat treatment can prevent salmonella contamination of feed ingredients to be transmitted to the clean areas (after heat treatment) of the feed mills and thereby to the compounded feed, although factors like the design of the feed mills and contamination from other external environmental sources can influence the result. It is logical to assume that when the salmonella contamination in incoming feed ingredients increase to a certain level, the feed mill environment may become contaminated which increases the risk for the contamination of the compounded feed. It is therefore also logical to assume that the feed mills of Company A, in spite of decontamination by organic acids, could not manage the relatively high level of salmonella contamination. It is also plausible to consider that this is the underlying reason why salmonella was transmitted to pigs fed by feed produced by its feed mills [8,9].

The feed ingredients found salmonella positive were treated by organic acids for decontamination purposes. This is a method which can reduce the contamination of salmonella [17] although the method might mask the detection of surviving microbes [18]. Even though the acid treated consignments were not allowed to enter feed mill until a negative test procedure, the decontamination process cannot be considered as a guarantee for freedom from salmonella but instead that the level of contamination has been brought under the detection level of the test method applied. The Swedish legislation therefore prescribes that feed including feed ingredients that have undergone decontamination by other methods than heat treatment must be heat treated. Suitable technical equipment and the layout of the production line is also essential for production of safe feed. Aspiration of dust is essential in all feed production and particularly when contaminated ingredients are introduced in the mill. The importance of a continuous monitoring for salmonella contamination of the feed production within a HACCP designed control, and the implementation of associated corrective actions when such contamination is detected, is in Sweden found to be of basic importance to prevent the introduction of salmonella into the food animal production and subsequently to the food chain [4]. Although the design of the feed mill may influence the number of samples required for an effective HACCP-surveillance, this study interestingly also found (data not shown) that in all the feed mills the in house monitoring for poultry feed was generally more intensive than was legally required. One Company (B) applied a more intensive heat treatment for poultry feed and in Company A up to 14 weekly environmental samples were taken in the poultry feed production, close to three times more than those five samples which are the minimum legal requirement. Long term documentation also demonstrates that the poultry feed (including feed from Company A) by the methods applied to prevent salmonella contamination, since the mid 1980-ies is virtually free from salmonella. The latter is indicated by the very low incidence of salmonella in the broiler production when each flock is tested before slaughter [1]. In Sweden the latter control, which on a voluntary basis started 1970, became mandatory 1984 in response to the spread of salmonella by feed [19,20]. It is thus justified to recommend that feed to all food animal species is treated equally.

The reason why so many as 28 serovars during a two year period were isolated from imported vegetable proteins is unknown. One explanation is that it can be the result of previous incidences contaminations of the crushing plants which not were eliminated but instead has been established as an in house contamination that can pop up periodically. Experiences from Denmark have demonstrated that in some crushing plants certain serovars of salmonella may persist for several years in the premises [21]. Similar experiences are gained in Sweden, e.g. the feed mill of Company B (Table 3).

Largely, the same serovars which were isolated from the feed ingredients, were re isolated in the monitoring of the feed mills (results not shown) thus demonstrating the spread of the contamination from the feed ingredients to the feed mills. If the feed mills cannot eliminate this contamination or ensure that it is not contaminating the compounded feed, a further spread to the food animals will occur which initiated this study [8,9]. At present there is a similar outbreak in the Southern part of Sweden where *S*. Typhimurium is considered to have spread from feed to animals, to the environment, to food and also to humans (Lahti 2009; personal communication).

Animals are thus infected per orally by salmonella contaminated feed in the same way as humans are infected by salmonella contaminated food. If the feed ingredients also include serovars which are known to have a preference for infecting humans as indicated by EFSA statistics [1] their occurrence in feed is also a threat to human health. In this study four (10.5%) of the 38 feed associated serovars isolated were included in the top ten list of isolates from human confirmed cases of salmonellosis in the EU [1]. In addition 76.3% out of the feed associated strains had been isolated from human cases of salmonellosis in Sweden. These finding require a further study and cannot be used a cause relationship with the salmonella isolated from feed ingredients and feed mills in this study. However, the results contradict the often used argument against the need for preventing salmonella contamination of feed by saying that feed associated serovars are considered usually to be non-pathogenic to humans Instead the result is in line with conclusions by EFSA that all serovars are potentially pathogenic to humans [5].

In summary there is a strong reason to prevent the introduction of salmonella into animal feed and efforts should focus on the crushing plants as the primary source for such introduction to the feed mills. The Scandinavian crushing plant mentioned in this study had not the capacity to supply all feed mills in Sweden. Some feed companies therefore have to buy from unknown sources with high risk for salmonella contamination like Company A of this study. The same challenging situation applies for whole EU due to the fact that 98% of soybeans or soybean meal is imported from third countries [10].

## Competing interests

The authors declare that they have no competing interests.

## Authors' contributions

The study and field work was designed, done and written by MW [20] based largely on industry laboratory data. Data from HACCP surveillance and from other official data from PH and from Swedish Board of Agriculture. Both authors revised the final manuscript.
